# Assessing the long-term health impact of Q-fever in the Netherlands: a prospective cohort study started in 2007 on the largest documented Q-fever outbreak to date

**DOI:** 10.1186/1471-2334-12-280

**Published:** 2012-10-30

**Authors:** Joris AF van Loenhout, W John Paget, Jan H Vercoulen, Clementine J Wijkmans, Jeannine LA Hautvast, Koos van der Velden

**Affiliations:** 1 Academic Collaborative Centre AMPHI, Department of Primary and Community Care, Radboud University Nijmegen Medical Centre, Nijmegen, the Netherlands; 2 Netherlands Institute for Health Services Research (NIVEL), Utrecht, the Netherlands; 3 Department of Medical Psychology, Radboud University Nijmegen Medical Centre, Nijmegen, the Netherlands; 4 Department of Infectious Disease Control, Municipal Health Service Hart voor Brabant, ‘s-Hertogenbosch, the Netherlands

**Keywords:** Q-fever, Coxiella burnetii, Health status, Quality of life, Legionnaires’ disease

## Abstract

**Background:**

Between 2007 and 2011, the Netherlands experienced the largest documented Q-fever outbreak to date with a total of 4108 notified acute Q-fever patients. Previous studies have indicated that Q-fever patients may suffer from long-lasting health effects, such as fatigue and reduced quality of life. Our study aims to determine the long-term health impact of Q-fever. It will also compare the health status of Q-fever patients with three reference groups: 1) healthy controls, 2) patients with Legionnaires’ disease and 3) persons with a Q-fever infection but a-specific symptoms.

**Methods/design:**

Two groups of Q-fever patients were included in a prospective cohort study. In the first group the onset of illness was in 2007–2008 and participation was at 12 and 48 months. In the second group the onset of illness was in 2010–2011 and participation was at 6 time intervals, from 3 to 24 months. The reference groups were included at only one time interval. The subjective health status, fatigue status and quality of life of patients will be assessed using two validated quality of life questionnaires.

**Discussion:**

This study is the largest prospective cohort study to date that focuses on the effects of acute Q-fever. It will determine the long-term (up to 4 years) health impact of Q-fever on patients and compare this to three different reference groups so that we can present a comprehensive assessment of disease progression over time.

## Background

Q-fever is a zoonosis that is caused by the intracellular bacterium *Coxiella burnetii*. A wide variety of animal species can be infected with the bacterium, including domesticated animals such as goats, sheep and cows [1]. Q-fever was known as an occupational illness until 2007, mainly infecting farmers, veterinarians and laboratory workers. An obligatory notification for Q-fever patients was introduced in the Netherlands in 1978 and the mean number of patients was around 17 cases per annum [2]. However, starting in 2007 the number of new patients increased annually, reaching a total of 4108 notified acute Q-fever patients over the period 2007–2011, of which at least 24 persons (0.6%) died [2].

Whilst the number of Q-fever cases has gradually increased in Europe in recent years, no other country has experienced an epidemic of this scale and the Dutch outbreak has become the largest documented outbreak in the world [3-13]. In comparison, the highest number of patients reported in an outbreak before 2007 was 415 in Switzerland [5]. Several measures were taken by the Dutch government in late 2009 to prevent the further spread of the disease (including the vaccination of goats and sheep on farms with more than 50 animals, and the culling of pregnant goats and sheep at farms that were found to be infected [14]), which has led to a massive reduction in the number of new patients since 2010 (there were 74 cases in 2011) [2].

Approximately 40% of all persons infected with Q-fever develop symptoms such as fever, pneumonia and hepatitis [5,15]. Several studies have shown that a relatively large group of patients suffer from persistent fatigue after acute Q-fever [16-18]. One study showed that the percentage of patients affected by persistent fatigue declines over time, ranging from 80% several weeks after infection, to less than 30% one year after onset of illness [17]. In another study, 42% of Q-fever patients reported symptoms fulfilling the criteria of Chronic Fatigue Syndrome one year after onset of illness [16]. This illness is also known as Post Q-fever Fatigue Syndrome, of which symptoms can last for as long as 10 years [18]. Among patients with an acute infection, an estimated 1.9% of patients develop chronic Q-fever, a potentially life-threatening condition [19]. Due to the limited number of large outbreaks to date, data from these studies are often based on a small numbers of patients which limits the accuracy and generalisability of the estimates. The current Q-fever outbreak in the Netherlands offers a unique opportunity to study the natural history of Q-fever infections, including the long term health impact, and ECDC has recommended the initiation of prospective cohort studies to better diagnose and treat acute Q-fever [19].

A number of studies have been conducted in the Netherlands that focus on Q-fever patients with an onset of illness in 2007/2008 and have monitored long-term symptoms (including fatigue), functional impairment and quality of life [20,21]. In a case–control study, Limonard et al. found severe fatigue levels in 52% of patients (N = 54), one year after onset of illness. In a retrospective study (N = 515), Morroy et al. found that 45% and 44% of patients were severely affected in their quality of life and fatigue respectively at least one year after they became ill. Both studies indicate that Q-fever causes serious long-term health problems. Limitations of these studies were 1) the health status of patients was only measured at 12 months after onset of illness, 2) the healthy control groups were small and not age-matched to the Q-fever patients, and 3) the health status of Q-fever patients was not compared to that of patients with another infectious illness [20,21].

Both Limonard et al. and Morroy et al. used the Nijmegen Clinical Screening Instrument (NCSI) to assess the health impact of acute Q-fever. This instrument was published in 2009 and combines a number of different health questionnaires. It was originally developed as a screening tool to assess the health status of patients with Chronic Obstructive Pulmonary Disease (COPD) [22], but has since been used in Q-fever studies [20,21]. The NCSI measures eight aspects of health status, covering symptoms (including fatigue), functional impairment and quality of life. Another instrument for measuring quality of life is the widely used Short Form 36 (SF-36), which was developed in 1988 by Ware and Stewart for the Medical Outcomes Study, and aims to correctly assess the quality of life of patients with a limited number of questions [23]. To our knowledge, the NCSI and SF-36 have never before been used side by side.

Apart from an Australian study which compared the long-term health impact of Q-fever patients to the health impact of patients infected with Epstein-Barr virus and Ross River virus [17], we have not found any other study that has compared the health impact of Q-fever patients to the health impact of patients with another infectious disease. This type of study is important as it can provide useful information on whether long-term health effects are specific to a certain infection, or whether they are common after suffering from any severe infectious illness. Patients suffering from Legionnaires’ disease were included as a reference group, as 1) the clinical manifestation of the acute phase of Legionnaires’ disease is similar to the acute phase of many of the Q-fever patients (almost all patients with Legionnaires’ disease and many Q-fever patients suffer from pneumonia), 2) the pathogens causing Legionnaires’ disease (Legionella pneumophila) and Q-fever (Coxiella burnetii) belong to the same order of Proteobacteria known as Legionellales [24] and 3) there is limited information on the long-term health impact of Legionnaires’ disease [25-27].

Not everyone who becomes infected with Q-fever becomes a notifiable Q-fever patient, as the majority of infected persons suffer from mild symptoms or no symptoms at all [5]. In this paper, we refer to this group as being *persons with a Q-fever infection but a-specific symptoms* and it is currently unknown whether they are at risk of developing persistent fatigue or chronic Q-fever. Our study has therefore included a group of persons with a-specific symptoms as an additional reference group.

## Methods/design

The designs used in this study are:

a prospective cohort study of Q-fever patients: one group of patients over a period of 2 years and another group over a period of 4 years;

three cross-sectional surveys of healthy controls, patients with Legionnaires’ disease 12 months after infection and persons with a Q-fever infection but a-specific symptoms roughly 4 years after infection.

**Figure 1:**
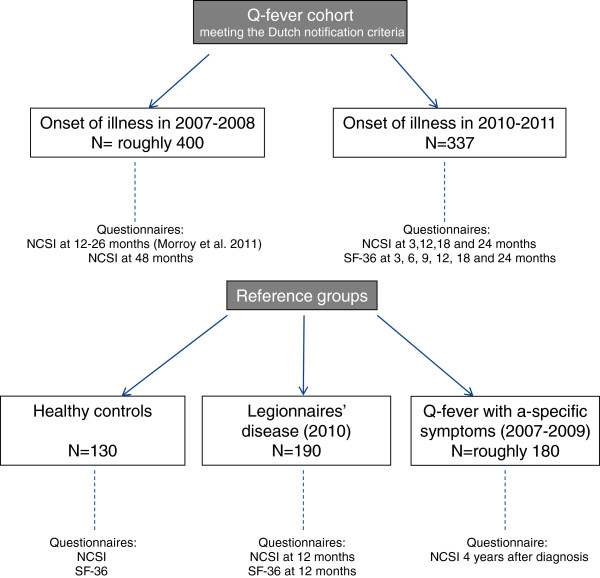
Explanation of the cohorts and reference groups and the timing of the questionnaires used.

All patients included in this study lived in the Netherlands, and were contacted between 2010 and 2013. The study protocol was submitted to the Medical Ethical Review Board of the Radboud University Nijmegen Medical Centre, which indicated that ethical review was not required as participation consists of filling out one or more anonymous questionnaires. The data on the healthy controls were derived from a different study, for which approval was given by the Medical Ethical Review Board of the Radboud University Nijmegen Medical Centre (reference number: 2006/243). Figure 1 shows which questionnaires were completed and at which time interval.

### Study population

#### Patients with Q-fever

Our first cohort group consisted of patients with an onset of illness in 2007 and 2008 from two Municipal Health Service regions in the province of Noord-Brabant who participated in the study of Morroy et al. [21]. Patients from that study who also gave permission to be included in further research studies were contacted by our researcher approximately 4 years after onset of illness. All patients fitted the Dutch notification criteria. This includes a positive serology by one of the following laboratory tests:

Identifying a seroconversion or a quadrupled or higher increase in IgG antibody titre against C. burnetti in a paired serum sample (sera obtained in the acute phase and recovery phase with a time interval ≥ 2 weeks) by indirect immunofluorescence or complement fixation test;

Presence of IgM-antibodies against phase II of C. burnetii;

Identifying C. burnetii by PCR or culture in blood or respiratory material;

Presence of antibodies against phase I of C. burnetii (chronic infection).

Until July 2008 a further requirement was a clinical presentation matching acute Q-fever. As of July 2008, this was refined and patients meeting the Dutch notification criteria had to have at least fever, pneumonia or hepatitis [28].

Patients diagnosed with Q-fever in 2010 and 2011 in the Netherlands, who were at least 18 years of age and fulfilled the Dutch notification criteria of Q-fever, were eligible for this prospective cohort. The notification criteria since 2010 include also an onset of illness within the previous 90 days. Eligible patients were informed about the study by the Municipal Health Service and after having received written consent, patients were included in the study.

#### Healthy controls

A control group consisting of healthy participants was formed by recruiting persons via advertisements in local newspapers in the city of Nijmegen area. The healthy controls were age-matched to the group of Q-fever patients and were asked to visit Radboud University Nijmegen Medical Centre, University Centre for Chronic Diseases Dekkerswald, where they completed electronic questionnaires (the NCSI and SF-36). The lung function of healthy controls was also tested, so that persons with an undiagnosed underlying illness that could affect their health status could be excluded.

#### Patients with Legionnaires’ disease

Patients diagnosed with Legionnaires’ disease according to the Dutch notification criteria and an onset of illness in 2010 were eligible to participate in this study. The Dutch notification criteria contain a case definition of an infection confirmed by at least one but preferably two of the following laboratory diagnostic tests:

Isolation of Legionella-species from respiratory secretions of blood;

Identification of the L. pneumophila-antigen in urine either by radio-immuno-assay or enzyme linked immunosorbent assay or immunochromatographic assay;

Identification of the Legionella-species by PCR in clinical material;

Identification of a significant titre of IgM-antibodies against L. pneumophila by ELISA;

Identification of a significant titre elevation of antibodies against L. pneumophila.

Further requirements are matching clinical symptoms, usually pneumonia [2]. Patients were recruited through the Municipal Health Services. Of the roughly 400 patients with an onset of illness in 2010, a sub-sample of these patients were contacted for our study (N = 243).

#### Persons with a Q-fever infection but a-specific symptoms

The last reference group consists of persons with a Q-fever infection but a-specific symptoms. Patients who tested positive for Q-fever and were notified by the laboratory to the Municipal Health Service, but who did not have symptoms fulfilling the Dutch notification criteria (fever, pneumonia or hepatitis), were eligible for this study group. Consent was obtained through the patient’s General Practitioner.

### Data collection

All patients were contacted by postal mail at determined time intervals after the onsets of illness (Figure 1). They received an information letter, a consent form and a questionnaire. Patients were asked to either return the signed consent form and the questionnaire simultaneously, or only the consent form stating that they did not want to participate. Patients who did not respond received a reminder by telephone or postal mail. Patients who returned an incomplete questionnaire were contacted by telephone by a member of the research team.

Only those Q-fever patients who reported severe fatigue and/or a severe impact on their quality of life at intervals 12 and 18 months after onset of illness were eligible to participate at intervals 18 and 24 respectively.

Since the onset of illness could not be determined for persons with a Q-fever infection but a-specific symptoms, these persons will be contacted approximately 4 years after their positive serology was confirmed by the laboratory. Persons with an onset of infection in 2007 and 2008 will be contacted in 2012, persons with an onset of infection in 2009 will be contacted in 2013.

### Questionnaire

Questionnaires were developed for the patients and reference groups in our study. The first questionnaire that patients receive collects information on risk factors for long-term impaired health status and symptoms due to Q-fever. The risk factors collected include age, smoking behaviour, alcohol consumption, education, Body Mass Index, pre-existing health problems (e.g. immune deficiencies, cancer, diabetes) and hospitalisation. Symptoms include all health effects that could be caused by Q-fever. Questions which may change over the course of the study are repeated in successive questionnaires (e.g. smoking behaviour, Body Mass Index, hospitalisation and symptoms). The NCSI and the SF-36 were used to measure aspects of health status, fatigue and quality of life. The NCSI [22] was included only every 6 months as it is longer and has not been tested at short time intervals (Figure 1). The SF-36 used was the official Dutch translation obtained from Quality Metric, Lincoln RI, USA. The NCSI and SF-36 were used simultaneously since they gather information on different domains.

### Data analysis

The main outcome measures are the health status, fatigue and quality of life of Q-fever patients at different intervals. A secondary outcome is the health status of Q-fever patients compared to the health status of healthy controls and patients with Legionnaires’ disease. Data will be analysed using the software SPSS for windows (version 18).

## Discussion

This study is the largest prospective cohort study that focuses on the long-term health effects of acute Q-fever to date. It will provide more insight into the short- and long-term (up to 4 years) health status, fatigue and quality of life of acute Q-fever patients. By comparing the long-term health effects to three reference groups (healthy persons, persons with a similar infectious disease (Legionnaires’ disease) and persons with a Q-fever infection but a-specific symptoms), a more comprehensive assessment of disease progression is better presented.

Even though there has been a major decline in the number of new acute Q-fever patients during the last few years, the disease still requires attention. A recent study in the Netherlands suggests that only a fraction (7.9%) of all Q-fever infections are notified to the public health authorities [29]. This is caused by a variety of factors, such as lack of clinical symptoms during the acute phase of the disease, not seeking medical attention, and not being tested for Q-fever with a diagnostic laboratory test. Assuming this percentage, the total number of infections in the Netherlands between 2007 and 2011 would be about 52000. Apart from the risks that are posed by chronic Q-fever, roughly 50% of persons that were diagnosed one or more years ago still suffer from long-lasting health effects such as severe fatigue or Post Q-fever Fatigue Syndrome, although the exact numbers are currently unknown [20,21]. Attention in the Netherlands is therefore now shifting from limiting the number of new infections to monitoring the long-term effects of acute Q-fever and providing support to these patients [19,29,30].

The findings of our study will be used by general practitioners and medical specialists to plan and organize the care for new and existing Q-fever patients, especially those with long-term symptoms. In addition, as a new Q-fever outbreak could occur in the Netherlands, elsewhere in Europe or internationally, it is important to assess and present the long-term health impact of this zoonotic infection.

Our study started data collection in September 2010 and will continue until the beginning of 2013. Data analysis will start in 2013 and results are expected in 2013 and 2014.

## Competing interests

The authors declare that they have no competing interests.

## Authors’ contributions

JAFvL participated in the design of the study, collected data and drafted the manuscript. WJP and JLAH participated in the design of the study and helped to draft the manuscript. JHV, CJW and KvdV participated in the design of the study and revised the manuscript. All authors read and approved the final manuscript.

## Pre-publication history

The pre-publication history for this paper can be accessed here:

http://www.biomedcentral.com/1471-2334/12/280/prepub
